# Morphometric Evaluation of Spermatogenic Cells and Seminiferous Tubules and Exploration of Luteinizing Hormone Beta Polypeptide in Testis of Datong Yak

**DOI:** 10.3390/ani10010066

**Published:** 2019-12-30

**Authors:** Qudratullah Kalwar, Min Chu, Anum Ali Ahmad, Xuezhi Ding, Xiaoyun Wu, Pengjia Bao, Ping Yan

**Affiliations:** 1 Key Laboratory of Yak Breeding Engineering, Lanzhou Institute of Husbandry and Pharmaceutical Sciences, Chinese Academy of Agricultural Science, Lanzhou 730050, China; qudratullahkalwar@gmail.com (Q.K.); chumin@caas.cn (M.C.); anum2017@lzu.edu.cn (A.A.A.); dingxuezhi@caas.cn (X.D.); wuxiaoyun@caas.cn (X.W.); baopengjia@caas.cn (P.B.); 2 Department of Animal Reproduction Shaheed Benazir Bhutto, University of Veterinary and Animal Sciences, Sakrand 67210, Pakistan; 3 State Key Laboratory of Grassland Agro Ecosystems, School of Life Sciences, Lanzhou University, Lanzhou 730050, China

**Keywords:** histomorphology, gene expression, *LHB*, yak, testis

## Abstract

**Simple Summary:**

Previous studies revealed that *luteinizing hormone βeta polypeptide* (*LHB*) plays an essential role in fertilization. Therefore, we aimed to confirm the importance of *LHB* in the testis of yak and to determine their association with male yak fertility. Histomorphological analysis of the testes is essential for predicting the fertilizing ability of the bull. To the best our knowledge, this is the first study to evaluate the micro anatomical changes and histometric alternation in testes of Datong yak. These findings could help to predict the sperm production capacity and to understand the specific molecular mechanisms of *LHB* during spermatogenesis.

**Abstract:**

Histological examination of testes is essential for understanding infertility, sex development, and growth. Therefore, to understand the histomorphology of testes at different developmental stages, we performed hematoxylin and eosin staining of Yak testis. Our results revealed that the diameters of spermatogenic cells and their nuclei were significantly larger (*p* < 0.05) in the testis at six years compared to at six and 18 months. No significant difference was noted between 30 months and six years. The study was designed to compare the expression profile of *LHB* in Datong yak. The expression pattern of *LHB* was explored using quantitative PCR, semi-quantitative PCR, molecular bioinformatic, and Western blot analysis. Our observations indicated that expression of *LHB* was significantly higher (*p* < 0.05) in the testis of Datong yak. Western blotting indicated that the molecular mass of LHB protein was 16 kDa in yak. The protein encoded by yak *LHB* included conserved cysteine-knot domain regions. The high expression of *LHB* in testis indicated that *LHB* may be vital for the development of male gonads and the fertility of Datong yak.

## 1. Introduction

The testes are vital organs in the male reproductive system that perform both exocrine (sperm secretion) and endocrine (testosterone hormone secretion) functions. Histologically, the testes are enclosed in a testicular tissue capsule through which blood vessels and nerves arrive and depart from the organ. The testis is composed of three layers: tunica vaginalis, tunica albuginea, and tunica vasculosa [1]. In certain animals, the smooth muscle cells in the testicular capsule support the movement of immovable spermatozoa into the duct system [2]. The testicular capsule has been found to respond, via contraction, to numerous electrical and chemical stimuli [3]. Peri tubular tissue (tunica or lamina propria) that lines the seminiferous tubules is present in all mammals [4]. The quantitative histology of the testicles, efficiency of Leydig and Sertoli cells, and general morphometry of the seminiferous tubule may be related to the quantity of spermatozoa in the ejaculate, as well as reproductive efficiency [5,6]. 

Luteinizing hormone βeta polypeptide (LHB) is a member of the beta chain family of glycoprotein hormones [7]. These glycoproteins are complex hormonally active macromolecules [8]. Luteinizing hormone is also known as lutropin subunit β (LHβ) and is vital for the maturation of the gonad and postnatal development of males [9]. Pituitary luteinizing hormone and placental chorionic gonadotropin stimulate luteinizing hormone receptors, which control steroidogenesis and the development of Leydig cells during, before, and after birth [10]. The β subunits, which originate from a gene complex on chromosome 19q13.32.2 during fetal life, result in chorionic gonadotropin initiating the growth of primordial Leydig cells and testosterone production, which in turn permits fetal masculinization [11]. Any alterations occurring in the luteinizing hormone receptor affect chorionic gonadotropin signaling in a male fetus, which results in clinical disorders ranging from undervirilized genitalia to complete pseudohermaphroditism [12,13]. Higher fertility and sexual maturation require normal testicular development, which is governed by chorionic gonadotropin in the uterus and after that by luteinizing hormone (LH) and follicle stimulating hormone (FSH) [14]. Most infertility occurs due to disturbance in the secretion of pituitary gonadotropic hormones or hypothalamic gonadotropin releasing hormone caused by a hypogonadotropic hypogonadism disorder [15]. The genetic changes that affect the signaling of gonadotropic hormones or their interactions with receptors may decrease fertility and affect sexual maturation [16]. 

The yak is one of the few animals that can survive the harsh environment of the Qinghai Tibetan Plateau [17]. However, low feed conversion efficiency, slow growth rate, and low production and reproductive performance are the main limitations to yak survival. *LHB* plays an essential role in the mammalian fertilization process [18]. Another study speculated that LH helps in ovulation, spermatogenesis, and the stimulation of ovaries and testis to synthesize steroid hormones [7]. However, all these findings were observed in other species, but no information has been published related to histomorphological changes and expression profiling of *LHB* in testis of yak at different ages. 

*LHB* characterization and understanding its function in the growth stages of yak may be important. To understand the role of *LHB* and its associations with the proteins, expression, and male fertility in yak, we explored the protein structure of bovine *LHB* using different bioinformatics methods and examined the expression profiling of *LHB* in Datong yak. The findings provided useful information about the reproduction of yak in understanding the molecular biology underlying animal fertilization and contributed to the understanding of exact gene functions related to yak reproduction.

## 2. Materials and Methods

### 2.1. Animals

This research was conducted at the Key Laboratory of Yak Breeding Engineering of Gansu Province, Lanzhou Institute of Husbandry and Pharmaceutical Sciences (Lanzhou, China). All yaks were handled in strict accordance with good animal practices that complied with the Animal Ethics Procedures and Guidelines of the People’s Republic of China. Only those animals that were in good health and experiencing no reproductive problems were included in this study. Samples were collected in strict accordance with the Guide for the Care and Use of Laboratory Animals, Lanzhou Institute of Husbandry Animal and Pharmaceutical Sciences, Lanzhou, China. Each animal was humanely slaughtered and all necessary efforts were made to minimize the risk of suffering. The legal certificate number was SCXK (Gan) 2014-0002. The yaks were classified into different age groups based on sexual activities: 6 months (male yak start to show mounting behavior, but not sexually mature), 18 months (sexual maturity of yak, but still have not started mating), 30 months (age at which yak start mating), and 6 years (peak mating age). Each age group contained four male yaks. The tissues collected from each animal included: intramuscular fat, spleen, heart, lung, kidney, liver, and testis. The extracted samples were frozen in liquid nitrogen for transportation and finally stored at −80 °C.

### 2.2. Exploration through Histology 

The testicle samples were maintained in 4% neutral buffered formalin for fixation overnight and later embedded in paraffin wax. The sections were cut into 5 µm slices, and then, sections were dehydrated in alcohol grading series (75–100%) for 2 min in each alcohol grade, as described by Taotao et al. [19]. Then, these sections were stained with hematoxylin and eosin (H&E): Harry’s hematoxylin for 2 min and 1% eosin for 30 s. Light microscopy analysis was conducted using an Olympus microscope (BX53) and camera (Olympus DP73, Tokyo, Japan). For each animal, 25 cross-sections of the most circular seminiferous tubules were photographed (10× and 20× objective lenses); in each section, the diameter and radius were measured. The mean value of two seminiferous epithelium heights was obtained by measuring their orthogonal positions. For Leydig cells, 10 sections per sample were examined by using ImageJ analysis software and a 100× objective lens.

### 2.3. Extraction of RNA and Synthesis of cDNA 

The whole RNA from each tissue was extracted using TRIzol reagent (TriPure Isolation Reagent, Roche, Carlsbad, CA, USA), following the manufacturer’s protocol. After total RNA extraction, the concentration, purity, and integrity of RNA were determined by Nanodrop (Thermo Fisher Scientific, Wilmington, DE, USA) and the Agilent 2100 Bioanalyzer (Agilent Technologies, Santa Clara, CA, USA), respectively. The complementary DNA (cDNA) was synthesized from 500 ng total RNA by the Prime Script^TM^ RT reagent kit with the gDNA Eraser Perfect Real Time (TaKaRa Bio Inc., Shiga, Japan). The prime script 1st strand cDNA synthesis kit (TaKaRa Bio Inc., Shiga, Japan) was used for cloning the bovine *LHB* sequence from the cDNA product and was reverse transcribed for 30 min at 42 °C, followed by incubation for 5 min at 65 °C. However, for semi-quantitative analysis, the cDNA template for reverse transcription reactions was performed at 37 °C for 15 min, followed by incubation at 85 °C for 5 s.

### 2.4. PCR Amplification and Primer Design

The National Center for Biotechnology Information (NCBI) was used for designing the primers for the gene expression and cloning (Table 1). The PCR was performed in a 25 μL volume containing 8.5 μL RNA-free H_2_O, 2 μL cDNA, 12.5 μL GoTaq green master mix, and 2 μL (10 pmol) primers (Promega, Madison, WI, USA). Thermal cyclic reaction was conducted for 2 min at 95 °C as the initial denaturation step followed by 35 cycles of denaturation for 1 min at 95 °C, annealing for 1 min at 58 to 61 °C, extension at 72 °C for 1 min, and a final extension at 72 °C for 5 min with holding at 4 °C. The amplification of primers was tested using 1.5% agarose gel and further confirmed by quantitative PCR (qPCR) by detecting the melting curve.

### 2.5. Cloning

The cloning was conducted in a 50 µL PCR, which included 1 µL (50 ng) cDNA, 1 µL (10 μM) each of reverse and forward primers, 25 µL of one-shot LA PCR^TM^ Mix (TaKaRa bio Inc., Shiga, Japan), and 28 µL dH_2_O. A cycling of the PCR was performed at 95 °C for 1 min; followed by 35 cycles of 95 °C for 10 s, at 61 °C for 15 min (annealing), 72 °C for 60 s (initial extension), and 15 min at 72 °C (final extension). The product reactions and the target bands were separated using 1% agar gel and using a gel extraction kit (Tiangen Biotech, Beijing, China), respectively. Lastly, the extracted product was cloned into the pMD19T vector and sequenced (TaKaRa Bio Inc., Shiga, Japan).

### 2.6. Analysis of LHB Using Quantitative Real-Time PCR

qPCR was used to determine the expression pattern of *LHB*. Glyceraldehyde 3 phosphate dehydrogenase (GAPDH) and beta actin (ACTB) were used as reference genes [20,21]. qPCR was performed with 12.5 µL TB Green^TM^ Premix Ex Taq^TM^ II (2×) (TaKaRa Bio Inc., Shiga, Japan). 10 pmol of primers, 2 µL DNA template (<100 ng), and 8.5 µL water. The Bio-Rad CFX96 Real Time Detection System (Bio-Rad, Hercules, CA, USA) was used for conducting the PCR at cycling conditions of 95 °C for 1 min followed by 39 cycles of denaturation at 95 °C for 10 s, for 30 s at 60 °C (annealing), and for 10 s at 68 °C (extension).

### 2.7. Analysis of LHB Using Semi-Quantitative PCR

The semi-quantitative PCR reactions for the analysis of *LHB* and *GAPDH* in Yak tissue samples were comprised of 162.5 µL Taq PCR Master Mix (Tiangen, Biotech, Beijing, China), 13 µL (10 μM)) each of forward and reverse primers, and 123.5 µL dH_2_O. The mixture was distributed into 24 µL aliquots in 10 tubes. Then, 1 µL (50 ng) of the cDNA template was added into each tube. PCR was completed using the thermal parameters for 5 min at 94 °C; followed by 35 cycles at 94 °C for 30 s, for 30 s at 56–61 °C (annealing), at 72 °C for 20 s, and for 5 min at 72 °C (final extension). The product reactions were determined on 1% agarose and imaged using ethidium bromide staining.

### 2.8. Bioinformatics Analysis

The conserved domains in the LHB protein were predicted using Pfam (http://pfam.janelia.org/). An open reading frame **(**ORF) finder program (http://www.ncbi.nlm.nih.gov/gorf/gorf.html) was used to calculate the sequence of amino acids from the coding sequence of *LHB.* PSI blast based secondary structure prediction (PSIPRED) (http://bioinf.cs.ucl.ac.uk/psipred) and Swiss Model (http://swissmodel.expasy.org/) was used for the determination of the three-dimensional (3D) and secondary structures of LHB.

### 2.9. Western Blot Analysis of LHB Protein

The protein was extracted from testicle samples, as described previously [22]. Firstly, samples from different groups were homogenized in ice-cold RIPA buffer (25 mM Tris/HCl (pH 7.6), 1% sodium deoxycholate, 150 mM NaCl, 1% Nonidet P40, and 0.05 Mm PMSF, 0.1% SDS) and centrifuged at 15,000× *g* for 10 min at 4 °C. Afterward, the total protein concentrations were measured with a commercial bicinchoninic acid Protein Assay kit (Beyotime, Shanghai, China). Then, samples (20 μg protein per lane) were subjected to electrophoresis on 12% Tricine SDS-PAGE and transferred onto PVDF membranes (Roche, Life Science, California, CA, USA). Then, 5% milk powder was used for blocking the layers in 1:9 PBS and for 60 min in 0.1% Tween 20 and incubation at 4 °C with primary antibodies (anti-LHB antibody and anti β-actin) overnight (diluted 1:1000; Abcam, Cambridge, UK). Then, membranes were incubated for 1 h with a goat anti-rabbit IgG (H+L) and horseradish peroxidase (HRP) conjugate (secondary antibody dilution in 1:5000; Transgen biotech, Beijing, China), and the results were imagined using an ECL detection system (GE Healthcare Bio-Sciences, Pittsburgh, PA, USA).

### 2.10. Statistical Analyses

The threshold cycle 2^−△△*C*t^ method was used to determine quantitative mRNA expression levels [23]. Results are presented as the mean ± the standard error of the mean (SEM). The histomorphometric analysis of stained tissue sections was measured using ImageJ software and analyzed statistically with Graph Pad Prism 7.0. We measured all areas and diameters based on the geometric constant “Pi” square root (A = π√2) (A = 3.14√2). The statistically significant differences among the protein levels and histomorphometric measures were also analyzed by one way ANOVA and the *t*-test (*p* < 0.05).

## 3. Results

### 3.1. Comparison of Morphological Differences in Different Yak Developmental Stages

**Figure 1:**
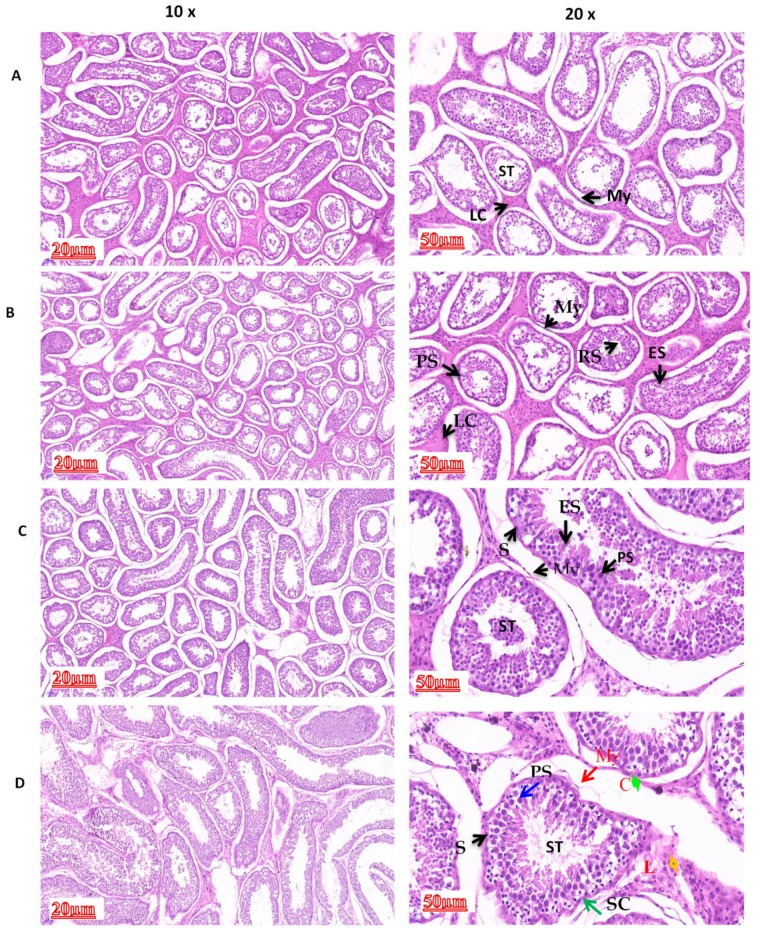
Morphological evaluations of testicular tissues at different ages: (**A**) 6, (**B**) 18, and (**C**) 30 months, and (**D**) 6 years. Different structures were found: myoid cell (My; red arrow), capillary (C), Leydig cell (L), spermatogonium (S; black arrowhead), Sertoli cell (SC; green arrow), primary spermatocyte (PS; Blue arrow), elongated spermatid (ES; Yellow arrow), Seminiferous tubule (ST). Scale bars = 20 µm (10×) and 50 µm (20×).

The histological findings obtained through H&E staining are presented in Figure 1. These results revealed that different structures, such as myoid cells, capillaries, Leydig cells, spermatogonia, Sertoli cells, primary spermatocytes, and round spermatid, were present in all ages (six years and 6, 18, and 30 months). We found no significant difference between six years and 30 months of age. However, cross-sectional area, epithelial thickness, and volume density of both seminiferous tubules and seminiferous epithelium increased gradually as the animal aged toward sexual maturity.

### 3.2. Diameters of Spermatogenic Cells and Their Nuclei in Yak Testis

The diameter of the spermatogenic cells and their nuclei in the testis of yaks at different growth stages increased from six months to six years (Table 2). The diameters of spermatogenic cells and their nuclei were significantly larger (*p* < 0.05) in the testis at six years compared to at six and 18 months. This difference was non-significant between 30 months and six years. The diameters of the spermatids in the different groups were non-significant (*p* > 0.05), whereas the diameters of the Leydig cells significantly increased (*p* < 0.05) as the yaks aged.

### 3.3. Diameter of the Seminiferous Tubule and Number of Cells in the Testicles

We found that the diameter of the seminiferous tubule significantly increased as yaks aged (Table 3). The seminiferous tubule diameters at 6 months, 18 months, 30 months, and 6 years were 190.0 ± 0.41, 220.82 ± 0.67, 235.69 ± 0.50, and 245.69 ± 0.59 μm, respectively. The tubules diameter between six and 18 month old yaks increased quickly before the onset of puberty in male yaks. The height of the seminiferous epithelium significantly increased (*p* < 0.01) from six months to six years in yaks. We found the increase in seminiferous epithelium height to be more obvious between six and 18 months of age. The seminiferous tubules volume density ranged from 68.21% ± 0.15% to 78.84% ± 0.73%. Our findings showed that luminal diameter, luminal area, Leydig cell area, and width of tunica albuginea increased as yaks aged. The mean cross-sectional area of the seminiferous tubules varied from 46,675.0 ± 2689 to 50,812 ± 3711 µm^2^ in different ages of yak, and the age groups were not significantly different. The total number of Sertoli cells, spermatogonium, spermatocyte, and Leydig cells per testis increased from six months to six years (Table 3), whereas the maximum numbers of these cells per testis were found in the six years age group, followed by the 30, 18, and 6 month age groups.

### 3.4. Expression Pattern of LHB Determined Using Semi-Quantitative PCR

**Figure 2:**
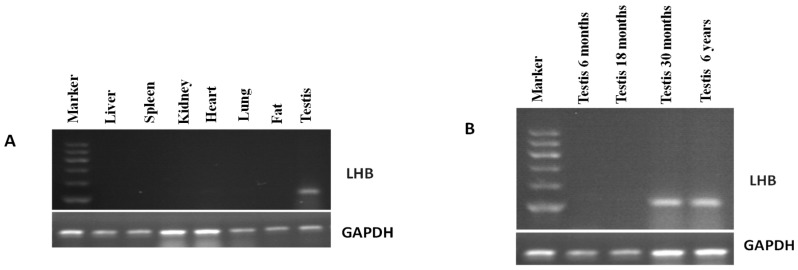
Analysis of the *LHB* mRNA using semi-quantitative PCR: (**A**) expression profiling from various yak tissues; (**B**) expression profiling at different yak growth stages.

In this study, we investigated the expression pattern of *LHB* in various tissues in yak using semi-quantitative PCR. The results showed that *LHB* was only expressed in testicles of yak, and not in any other tissues (Figure 2A). The comparison of testes from different ages of bulls revealed that the expression of *LHB* was prominent at six years and 30 months of age and was not observed at six or 18 months (Figure 2B). On the basis of these differences, we hypothesized that the higher expression of *LHB* in the testis indicated that *LHB* played a major role in the spermatogenesis and fertility of animals.

### 3.5. Expression Pattern of LHB Determined Using Quantitative Real-Time PCR

**Figure 3:**
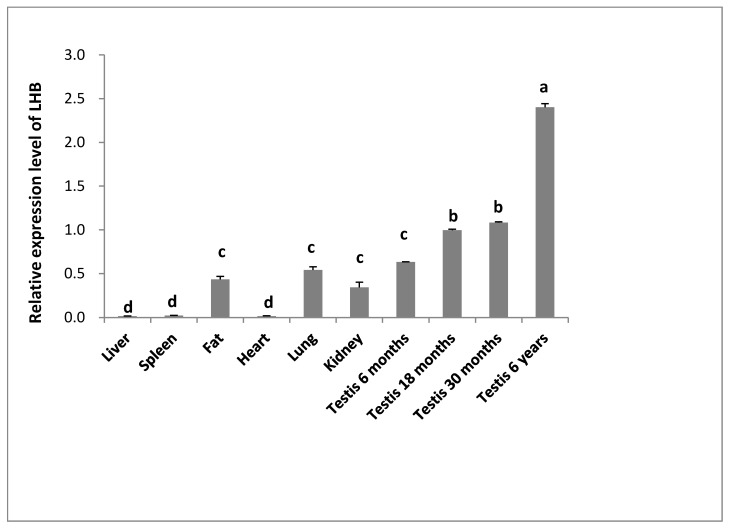
The relative expression of the *LHB* mRNA level was evaluated by quantitative real-time PCR from different tissues of Datong yak. Different letters indicate a significant difference (*p* < 0.05).

The expression profile of *LHB* mRNA was also evaluated by performing qPCR in various tissues of yak. These findings specified that the LHB mRNA was also present in other tissues, but the expression of LHB was significantly lower in other tissues as compared to testis. Moreover, moderate expression was detected in lungs followed by intramuscular and kidney, while lower expression was observed in heart, spleen, and liver (Figure 3).

### 3.6. Structures of LHB

**Figure 4:**
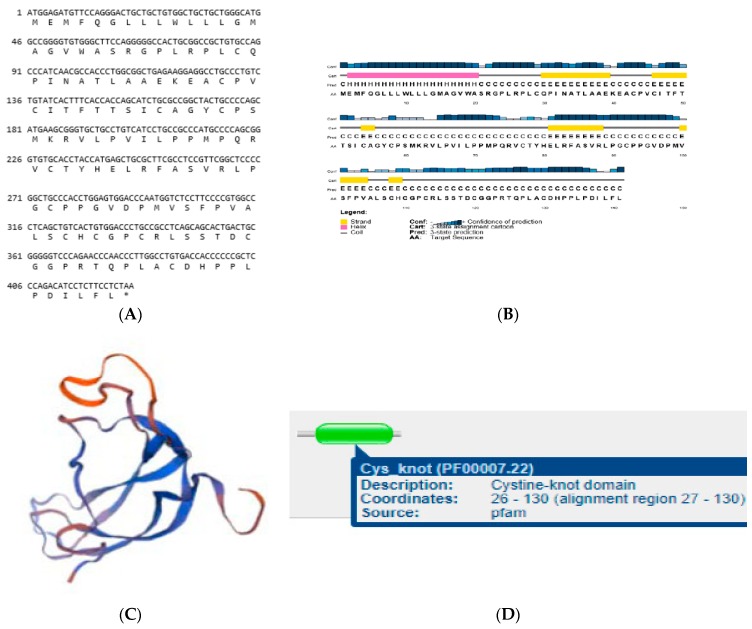
Assessment of the protein sequence encoded by *LHB* from the yak. The protein encoded by yak *LHB* contains a cysteine-knot domain. (**A**) The sequence of yak *LHB* and the predicted protein; (**B**) assessed secondary structures encoded by the LHB protein has a long vertical bar, short vertical bar, medium vertical bar, coil α-helix, extended strand, and sub-medium vertical bar turn; (**C**) estimated three-dimensional structures of LHB proteins; and (**D**) prediction of the conserved domain for LHB.

**Figure 5:**
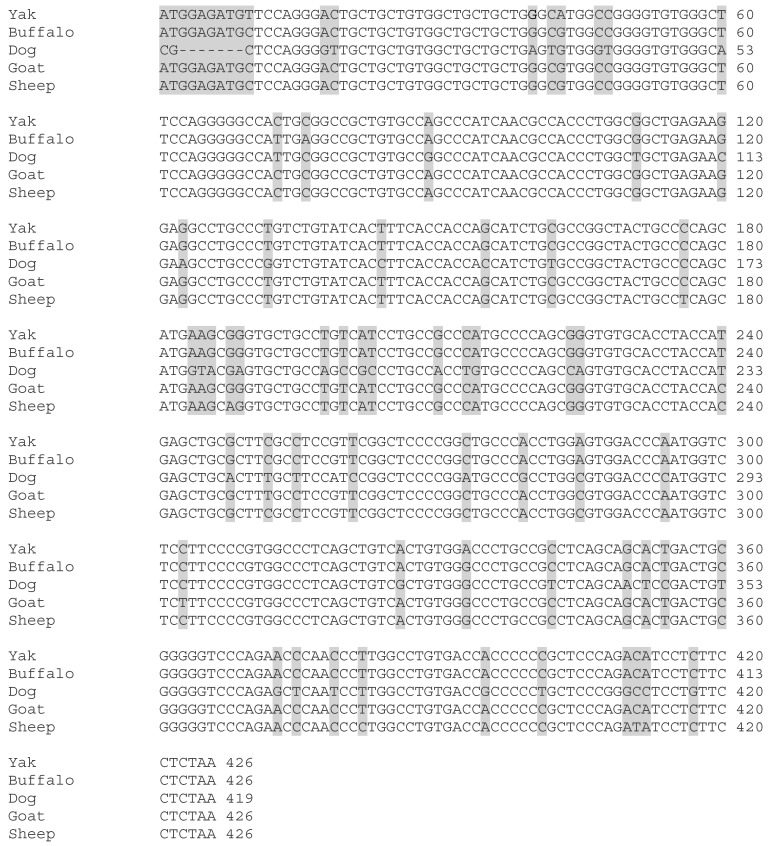
Multiple alignment of the full length sequence of *LHB* in different animals. Different amino acids are characterized by shaded boxes.

We cloned the coding regions of *LHB* from yak testis. Afterward, we used different bioinformatics tools to examine the protein coded by *LHB*, with an emphasis on the functional sites and secondary structures. The coding region sequences of *LHB* in yak encoded 141 amino acids (Figure 4A). The protein encoded by yak *LHB* contained a cysteine-knot domain (Figure 4D), and the secondary structures consisted of helixes, coils, and an extended strand (Figure 4B). The 3D structure of the LHB protein was determined to have a 1.9 Å resolution using SWISS-MODEL (Figure 4C). The multiple alignment sequence of the corresponding homologous regions shared a sequence homology of 100%, 99.30%, 85.51%, 97.50%, and 96.92% with yak, buffalo, dog, goat, and sheep, respectively (Figure 5). Thus, these results showed that LHB protein was extremely conserved between all mammals.

### 3.7. Western Blot Analysis

**Figure 6:**
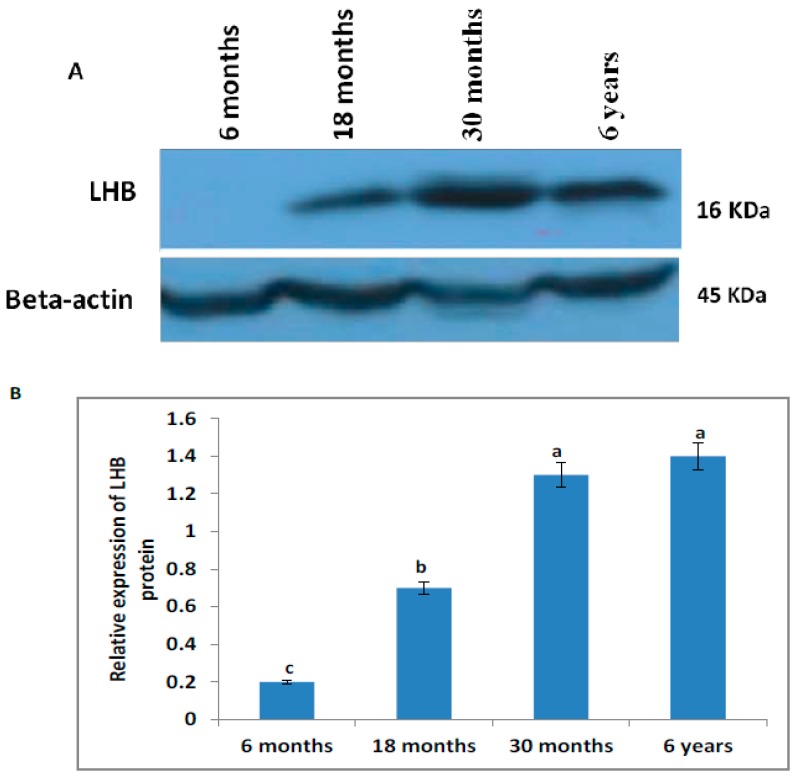
Detection of the expression pattern of LHB protein in Datong yak testis using Western blotting: (**A**) Western blot of LHB protein and (**B**) relative LHB protein expression levels were quantified by densitometric analysis. Protein levels were quantified by densitometric analysis. Different letters indicate significant difference (*p* < 0.05).

The characterization of LHB protein in the testis of different growth stages of yak was confirmed using Western blot analysis (Figure 6). The testes from the different groups showed no significant differences in the expression of LHB protein between 30 months and six years, but the expression levels in these two groups were significantly higher than at six or 18 months.

## 4. Discussion

Infertility is a serious problem in several livestock species, as well as in humans. Almost 25% of humans and 35% of animals produce live offspring, and 15% of couples are unable to conceive within one year of unprotected intercourse and remain childless due to infertility problems [24]. However, limited numbers of genetic causes of infertility have been recognized in animals, as well as in humans. More than 80 genes are involved in the fertility of humans and mice [24], and gonadotropins are the key endocrine regulators for the normal process of spermatogenesis [25]. During spermatogenesis, *LHB* targets the Leydig cells to enhance the secretion of androgens, i.e., testosterone, which in turn acts on androgen receptors in the seminiferous epithelium to control the process of spermatogenesis [26]. Another study revealed that reproduction mainly depends on the regulated expression of the *LH β* gene [27]. Therefore, we wanted to confirm the significance of *LHB* in different yak organs, and the results of the semi-quantitative PCR showed that the expression of LHB was higher in the testicles, but not detectable in any other tissue. This finding strongly supported the hypothesis that *LHB* was related to reproduction and sexual development in bovine animals. In a previous study, Ma et al. [18] determined that postnatal defects in gonadal growth resulted in infertility due to the targeted disruption of the *LHB* gene. Testis mutation results in decreased testis size, prominent Leydig cell hypoplasia, abnormalities in the expression of genes encoding steroid biosynthesis pathway enzymes, reduced testosterone levels, and blockage of spermatogenesis at the round spermatid stage. Yang et al. [13] recognized a novel mutation in the *LHB* gene in a male patient with hypogonadism, which indicated that *LHB* mutation can cause selective LH insufficiency, resulting in infertility.

The results of the quantitative PCR showed that *LHB* mRNA is expressed in other tissues of yak; however, its expression was significantly higher in the testicles of yak compared to the other tissues. Other studies reported that luteinizing hormone develops in the pituitary gland and plays a central role in promoting ovulation and spermatogenesis by stimulating the ovaries and testes to synthesize steroids [28]. The *LHB* sequence was analyzed for a 30-year-old patient who was suffering from hypogonadotropic hypogonadism, and the patient was treated with human chorionic gonadotropin (hCG), resulting in adequate spermatogenesis [29,30]. Similarly, in another study, Weiss et al. [31] reported a patient with biologically inactive LH homozygosity for a missense mutation in *LHB*, who had three maternal uncles who were infertile, but showed normal secondary sexual characteristics. The functional analysis confirmed that the mutation prevents LH from binding to its receptor. However, spermatogenesis is reduced in LH deficient men, ranging from azoospermia to oligospermia, which has been linked to the lack of LH stimulation and low intratesticular testosterone action [32].

Our findings show that the protein encoded by Datong yak *LHB* contained a cysteine-knot domain. Furthermore, another study stated that β-subunits belonged to the cysteine-knot superfamily and have tertiary structures analogous to three extended loops [33]. The C-terminal segment of the β subunit stabilizes the heterodimer by wrapping around the α subunit like a seat belt and latching through a disulfide bond with a cysteine residue from loop 1 of the β subunit [34,35]. Cysteine residues in amino acid chains are vital for disulfide bonding and the formation of loops to produce functional motifs in the tertiary structures of various proteins [36].

Our findings revealed that the seminiferous tubule diameter increased with advancing age. The seminiferous tubule diameter at 6, 18, and 30 months and six years was 190.0 ± 0.41, 220.82 ± 0.67, 225.69 ± 0.50, and 235.69 ± 0.59 µm, respectively. The significant increase in diameter between six and 18 months indicated fast development of the tubules before sexual maturity. Similar findings were documented in Assam goats [37] and cattle [38]. Our results indicated a highly significant (*p* < 0.05) increase in the diameter of the seminiferous tubules at different ages. Baishya et al. [39] stated that with increasing age, the diameter of the seminiferous tubule increased, but the rate of increase was not significant. Ahmad et al. [40] reported the seminiferous tubule diameter as 176.8 ± 2.6 µm in Nili-Ravi buffalo bulls at 24 months of age. Conversely, Akosman et al. [41] documented the diameter of seminiferous tubules as 223.44 µm and 226.68 µm in Holstein and Simmental bulls, respectively. Mohammed et al. [42] reported a larger seminiferous tubule diameter of 258 ± 1.9 µm in goats. Ibrahim et al. [43] reported that an increase in the process of spermatogenesis led to an increase in the thickness and diameter of seminiferous.

The seminiferous epithelium height increased significantly (*p* < 0.05) as yaks aged toward sexual maturity from six months to six years. The seminiferous epithelium height increased from six to 18 months of age, which is the period of puberty for yak. The current results were in agreement with those reported by Sarma and Devi [37] and Nishimura et al. [44] in Assam goats and male Tokara goats, respectively. In the current findings, seminiferous tubule volume density was recorded as ranging from 68.21% ± 0.15% to 78.84% ± 0.73%. Similarly, other studies revealed that volume densities ranged from 70% to 90% of the testis parenchyma in most mammalians [45]. Our findings were also in contrast with Sarma and Devi [36] who reported that different micro parameters of the seminiferous tubules increased with increasing male goat age, and they found a significant difference in growth between six and eight months. Our findings illustrated that luminal diameter, luminal area, Leydig cell area, and width of tunica albuginea increased as yaks aged toward sexual maturity. Sheeraz et al. [46] evaluated luminal diameter (69.10 ± 16.99 µm^2^), luminal area (49.26 ± 8.47 µm^2^), Leydig cell area (108.19 ± 30.60 µm^2^), and width of tunica albuginea (12.99 ± 1.35 µm) in mice. In our findings, the mean cross-sectional area of the seminiferous tubules varied from 46,675.0 ± 2689 to 50,812 ± 3711 µm^2^ for different ages of yak, and the differences were not significantly different. Our results were in agreement with those reported by Paulo et al. [47]; they found no significant differences in cross-sectional area of the seminiferous tubules, which ranged from 47,785.0 to 51,914.1 µm^2^ in different zebu bull breeds. The total number of spermatogonia, spermatocytes, Leydig cells, and Sertoli cells per testis increased from six months to six years (Table 2). The maximum numbers of these cells per testis was higher at six years of age followed by 30, 18, and then, 6 months of age. Our findings were consistent with those of Karmore et al. [48], who reported that the number of these cells was lower in prepubertal than in pubertal and post-pubertal animals. Sun et al. [49] reported that cattle yak had similar histomorphological structures at 10, 12, and 14 months of age. Another study showed that the seminiferous tubules of cattle yak primarily contained Sertoli cells and spermatogonia and were highly vacuolated, whereas those of cattle and yak contained abundant primary spermatocytes when three years old [50]. The expression of LHB protein in testis was also confirmed by Western blot analysis. The data showed that the expression of LHB protein in testis of yak had a 16 kDa molecular mass. Caroline et al. [51] detected a size of approximately 15 kDa in the wild-type and mutant luteinizing hormone β subunits in HEK 293T cells.

## 5. Conclusions

Our results revealed the high expression of *LHB* in the testicles of yak, which indicated that *LHB* might be essential for spermatogenesis and the synthesis of steroids and hormones. *LHB* can be used as a candidate gene for improving the fertility of yak during the non-breeding season. Our findings demonstrated that age significantly affected the micro anatomical aspects of yak. These findings may help anatomists, pathologists, and theriogenologists in predicting the fertilizing ability and sperm production capability of male breeding yaks.

## Figures and Tables

**Table 1:** Primers used for qPCR and cloning.

Accession No	Gene	Primer Sequence (5’- >3’)	Product Length (bp)	Annealing Temperature (°C)
XM_005895365.2	*LHB* (For cloning)	F: TGGAGTGGACCCAATGGTCT	426	60.48
		R:TAGGATGGGCATGGGAGGTT		60.33
XM_019980229.1	*LHB* (For gene expression)	F:ACCAGTGCAGTGAGACGAAG	131	59.97
		R:GATTAAAGCCTGGGAAGGGC		58.60
NM_001034034.2	*GAPDH*	F: ATGAAAGGGCCATCACCATC	204	55.85
		R: GTGGTTCACGCCCATCACA		60.00
L08165.1	*β-actin*	F:TGGGTATGGAGTCCTGTGGT	160	60.00
		R: AGGGCTGTGATCTCCTTCTG		60.40

F: forward primer; R: reverse primer; GAPDH: glyceraldehyde-3-phosphate dehydrogenase.

**Table 2:** Diameters (μm) of spermatogenic cells and their nuclei in the testis of yak (mean ± standard error of the mean (SEM).

Germ Cells	6 Months	18 Months	30 Months	6 Years
Spermatogonium	4.23 ± 0.56 ^a^	4.60 ± 0.38 ^a^	5.90 ± 0.70 ^b^	6.52 ± 0.79 ^b^
Spermatogonium nuclei	2.30 ± 1.20 ^a^	2.70 ± 1.80 ^a^	3.85 ± 1.20 ^b^	4.80 ± 1.80 ^b^
Primary spermatocyte	4.55 ± 0.41^a^	5.13 ± 0.50 ^a^	6.20 ± 0.90 ^b^	7.10 ± 0.89 ^b^
Primary spermatocyte nuclei	2.10 ± 0.98 ^a^	2.63 ± 1.76 ^a^	3.70 ± 0.98 ^b^	5.63 ± 1.76 ^b^
Round spermatid	4.90 ± 1.10 ^a^	5.22 ± 2.43 ^a^	6.10 ± 1.10 ^b^	8.22 ± 2.43 ^b^
Round spermatid nuclei	4.01 ± 1.21 ^a^	6.89 ± 1.72 ^ab^	7.01 ± 1.21 ^b^	6.89 ± 1.72 ^b^
Sertoli cells	2.30 ± 0.76 ^a^	3.17 ± 0.52 ^b^	4.70 ± 0.49 ^c^	5.20 ± 0.96 ^c^
Leydig cells	4.10 ± 0.66 ^a^	5.10 ± 1.76 ^b^	6.20 ± 1.65 ^c^	8.30 ± 1.52 ^d^

Note: Means with dissimilar superscripts in the same row are significantly different (*p* < 0.05).

**Table 3:** Diameter (μm) of the seminiferous tubule and the number of cells in the testicles of different ages of yak (mean ± SEM).

Germ Cells	6 Months	18 Months	30 Months	6 Years
Tubular diameter (μm)	190.0 ± 0.41 ^a^	220.82 ± 0.67 ^b^	235.69 ± 0.50 ^c^	245.69 ± 0.59 ^d^
Epithelial height (μm)	45.70 ± 0.45 ^a^	60.33 ± 0.75 ^b^	61.99 ± 0.67 ^b^	72.99 ± 0.52 ^c^
Luminal diameter (µm^2^)	50.23 ± 0.67 ^a^	60.94 ± 0.92 ^b^	72.84 ± 0.66 ^c^	88.94 ± 0.82 ^d^
Luminal area (µm^2^)	37.40 ± 9.38 ^a^	49.26 ± 8.47 ^b^	56.29 ± 6.70 ^c^	68.33 ± 8.244 ^d^
Leydig cell area (µm^2^)	90.19 ± 10.60 ^a^	114.73 ± 20.6 ^b^	126.99 ± 26.10 ^c^	149.76 ± 34.28 ^d^
Width of tunica Albuginea (µm)	12.09 ± 0.20 ^a^	13.05 ± 1.09 ^a^	14.30 ± 1.1 ^a^	15.41 ± 0.80 ^a^
Cross sectional area (µm^2^)	46,675.0 ± 2689 ^a^	47,695.0 ± 2910 ^a^	48,200 ± 3025 ^a^	50,812 ± 3711 ^a^
ST Volume density (%)	68.21 ± 0.15 ^a^	70.84 ± 0.73 ^a^	75.25 ± 0.50 ^a^	78.84 ± 0.73 ^b^
Leydig cells (%)	8.00 ± 3.56 ^a^	10.00 ± 1.76 ^b^	26.00 ± 2.65 ^a^	30.00 ± 1.52 ^a^
Sertoli cells (%)	16.20 ± 2.56 ^a^	20.0 ± 3.55 ^b^	26.00 ± 2.46 ^c^	41.00 ± 4.56 ^d^
Spermatogonium (%)	85.33 ± 17.60 ^a^	160 ± 25.49 ^b^	180 ± 15.80 ^c^	200 ± 18.79 ^d^
Spermatocyte (%)	9.00 ± 1.86 ^a^	14.60 ± 1.50 ^b^	17.25 ± 2.80 ^c^	20.50 ± 1.90 ^d^

Note: Means with different superscripts within the same row are significantly different (*p* < 0.05).
